# Inulin‐type fructans with different degrees of polymerization improve insulin resistance, metabolic parameters, and hormonal status in overweight and obese women with polycystic ovary syndrome: A randomized double‐blind, placebo‐controlled clinical trial

**DOI:** 10.1002/fsn3.3899

**Published:** 2023-12-26

**Authors:** Rahele Ziaei, Zahra Shahshahan, Hatav Ghasemi‐Tehrani, Zahra Heidari, Marilyn S. Nehls, Reza Ghiasvand

**Affiliations:** ^1^ Department of Community Nutrition, School of Nutrition and Food Science Isfahan University of Medical Sciences Isfahan Iran; ^2^ Department of Obstetrics and Gynecology, School of Medicine Isfahan University of Medical Sciences Isfahan Iran; ^3^ Fertility Department, School of Medicine Isfahan University of Medical Sciences Isfahan Iran; ^4^ Department of Biostatistics and Epidemiology, School of Health Isfahan University of Medical Sciences Isfahan Iran; ^5^ Department of Kinesiology and Health Promotion University of Kentucky Lexington Kentucky USA

**Keywords:** insulin, inulin, microbiota, obese, polycystic ovary syndrome, prebiotics

## Abstract

Polycystic ovary syndrome (PCOS) is associated with reproductive disorders and adverse cardiometabolic risk factors that can negatively impact the general health of women. Inulin‐type fructans (ITFs) are proposed to beneficially affect risk factors associated with metabolic disorders. Whether ITFs can help with the management of PCOS by modifying insulin resistance (IR) and androgen levels has not yet been explored. The aim of this study was to investigate the effects of ITFs with different degrees of polymerization on insulin resistance, blood lipids, anthropometric measures, and hormonal status in overweight and obese women with PCOS. In a randomized double‐blind placebo‐controlled trial, seventy‐five women with PCOS aged 18–40 years old were randomly assigned to receive 10 g/day of high‐performance inulin (HPI) or oligofructose‐enriched inulin (OEI) or maltodextrin for 12 weeks. Biochemical and clinical outcomes were measured at baseline and after the intervention. Participants in the HPI and OEI groups experienced improvements in waist circumference, total testosterone, free androgen index, sex hormone‐binding globulin, and triglycerides compared to the placebo group. Also, the number of women with irregular menses or oligomenorrhoea decreased significantly in both ITF groups. Participants in the HPI group reported lower body mass, fasting insulin, and HOMA‐IR, as well as a higher quantitative insulin sensitivity check index. ITF supplementation, especially with long‐chain ITFs, when given for 12 weeks may improve metabolic outcomes, androgen status and clinical manifestations in women with PCOS.

## Graphical abstract

Supplementation with inulin‐type fructans for 12 weeks improves weight loss, androgen status, and clinical manifestations of PCOS. The beneficial effects of high‐performance inulin on weight loss, insulin resistance, and hirsutism are more remarkable than those of oligofructose‐enriched inulin.

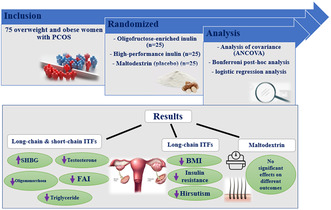

## 1 INTRODUCTION

Polycystic ovary syndrome (PCOS) is the most widespread endocrine disease in women of reproductive age, and it is believed to affect 6%–20% of women worldwide (Deswal et al., 2020). It is defined by the presence of at least two of three classic features: ovulatory dysfunction, clinical or biochemical hyperandrogenism, and polycystic ovaries (defined as 12 or more antral follicles (2–9 mm in diameter) in either ovary, an ovarian volume that is greater than 10 mL in either ovary, or both) (Rotterdam ESHRE/ASRM‐Sponsored PCOS Consensus Workshop Group, 2004). The most reliable clinical marker of androgen excess is hirsutism, defined as a modified Ferriman–Gallwey score ≥8. Ovulatory dysfunction mainly presents in the form of oligomenorrhea or amenorrhea. More rarely, oligoovulation leads to polymenorrhea. Polycystic ovary syndrome is associated with adverse cardiometabolic risk factors including insulin resistance (IR), obesity, metabolic syndrome, dyslipidemia, and hypertension, which can all negatively impact the general health of women (McCartney & Marshall, 2016; Osibogun et al., 2020).

The exact etiology of this complex disorder remains unclear; however, genetic, neuroendocrine, and nutritional factors are suggested to cause or increase the risk of PCOS (Azziz, 2018; Singh et al., 2023). Many studies have demonstrated the interconnection between PCOS and changes in the composition of the gut microbiota (He & Li, 2020; Rizk & Thackray, 2021). Increased intestinal mucosal permeability and leakage of lipopolysaccharides (LPS) into systemic circulation following gut dysbiosis lead to LPS‐induced endotoxemia (He & Li, 2020). The resulting low‐grade inflammation interferes with the function of insulin receptors, leading to IR and a compensatory hyperinsulinemia, which promotes androgen secretion by ovarian theca cells and interrupts normal follicular development (Sun et al., 2023).

Gut bacterial composition and diversity depend on many factors, including diet and the consumption of dietary fibers and prebiotics (He & Shi, 2017; Peredo‐Lovillo et al., 2020). A prebiotic is “a substrate that is selectively utilized by host microorganisms, conferring a health benefit” (Gibson et al., 2017). Inulin‐type fructans (ITFs), plant‐stored polysaccharides consisting of β‐(2‐1)‐linked fructose residues, are among the most studied prebiotic fibers that can promote the growth of beneficial gut bacteria (Nagy et al., 2022; Vandeputte et al., 2017). ITFs are mainly produced from chicory roots and can be separated into inulin [degree of polymerization (DP) 2–60], short‐chain fructooligosaccharides (DP 2–4), and oligofructose (DP < 10). The bifidogenic effects of ITFs have been well established, as ITFs increase the abundance of *Bifidobacterium* spp. Bifidobacterium are believed to have numerous health‐promoting effects, including the maintenance of gut epithelial integrity and modulating immune responses (Nagy et al., 2022). Evidence exists on the key role of gut microbiota in the development of IR in PCOS (He & Li, 2020). Inulin‐type fructans may improve metabolic endotoxemia, inflammation, and metabolic defects, including IR (Chambers et al., 2019; Tawfick et al., 2022), as well as help with the management of PCOS by modifying gut bacterial composition (Xue et al., 2019). Additionally, IR may be involved in the three classical features and metabolic defects associated with PCOS (He & Li, 2020; Zeng et al., 2020). So, targeting IR may be an effective approach in the management of PCOS. The beneficial effects of some prebiotics, such as resistant dextrin, on metabolic and hormonal status in women with PCOS have been proposed previously (Gholizadeh Shamasbi et al., 2019); however, to our knowledge, there are no studies on the effect of ITFs on IR and androgen levels in these patients.

When considering ITF supplementation, it should be noted that the prebiotic effects of ITFs depend on their DP (Du et al., 2020; Li et al., 2020). Whether ITFs of lower DP or higher DP exert more pronounced prebiotic effects in humans is not fully understood due to inconsistent findings (Astó et al., 2019).

Despite having several short‐term and long‐term health consequences, PCOS remains one of the most poorly understood medical disorders among scientists, patients, and physicians. Currently, there is no specific pharmacological treatment approved for PCOS by the FDA or other health authorities. Identifying dietary interventions that help to improve PCOS symptoms and complications by affecting IR is of great importance. We hypothesized that ITFs might be an effective and safe therapeutic approach in women with PCOS via the modification of IR and excess androgens. Thus, we conducted an intervention to investigate the impact of ITF supplementation on weight loss, IR, blood lipids, and biochemical and clinical hyperandrogenism in overweight and obese women with PCOS. Additionally, this study aimed to measure if ITFs with different DPs may have different effects on these outcomes.

## 2 METHODS AND MATERIALS

### 2.1 Trial design

The present study is a randomized, double‐blind, placebo‐controlled, parallel‐group clinical trial. The subjects were recruited from Shahid‐Beheshti Women's Hospital and The Gynecology Clinic at Amin Hospital, affiliated with the Isfahan University of Medical Sciences (IUMS), Isfahan, Iran, between October 2020 and March 2021. Women with PCOS, diagnosed using the 2003 Rotterdam criteria (Rotterdam ESHRE/ASRM‐Sponsored PCOS Consensus Workshop Group, 2004), aged between 18 and 40 years, and with a body mass index (BMI) between 25 and 35 kg/m^2^, were included in the study. Participants were provided with an information sheet explaining the protocol and the potential benefits and adverse effects of the intervention, and consent was obtained from all participants. The study protocol was approved by the Research Council (Approval No: 399461) and Ethics Committee (IR. MUI.RESEACH. REC.1399.471) of Isfahan University of Medical Sciences, Isfahan, Iran, and was registered at the Iranian Registry of Clinical Trials (IRCT, www.irct.ir) (Ref. No: IRCT20101101005062N11).

### 2.2 Participants

Through purposive sampling, women with PCOS admitted to two hospitals were invited to participate. We also advertised at the hospital or clinic lobby to invite participants. PCOS was diagnosed by two experienced gynecologists (Z.SH and H.T) following the 2003 Rotterdam criteria, and patients with two of the following were considered to have PCOS: biochemical indicators (serum total testosterone >0.481 ng/mL [conversion factor to SI unit (nmol/L): 3/467]) and/or clinical signs of hyperandrogenism (hirsutism using modified Ferriman–Gallwey score of ≥8), oligo‐ovulation and/or anovulation (menstrual cycle length less than 21 days or greater than 35 days), and polycystic ovaries on ultrasonography (12 or more antral follicles measuring 2–9 mm in diameter in each ovary and/or increased ovarian volume >10 mL^3^, in either ovary or both) (Rotterdam ESHRE/ASRM‐Sponsored PCOS Consensus Workshop Group, 2004). Participants were excluded if they: were younger than 18 years or older than 40 years; had BMI ≤25 or ≥ 35 kg/m^2^; were current or previous (within the last 3 months) users of oral contraceptive drugs, hormone therapy, weight‐loss interventions, probiotics, prebiotics or synbiotic supplements, anti‐acid drugs, antibiotics or multivitamin mineral supplements; had a history of autoimmune diseases, any type of cancer, cardiovascular diseases, abnormal liver function test, any type of kidney disease, other endocrine disorders including thyroid disorders, diabetes or impaired glucose tolerance, Cushing's syndrome, hyperprolactinemia and androgenic disorders, functional gastrointestinal diseases (all based on patients' medical records); were allergic to ITFs or placebo; were following specific diet or physical activity programs; were current smokers; were pregnant (or planning for pregnancy within next 6 months) or lactating; or had poor compliance (less than 80%) with the intervention (Armijo‐Olivo et al., 2009).

### 2.3 Intervention

Participants were randomized into two intervention groups and one placebo group (1:1:1). Two ITFs with different DPs were used. High‐performance inulin (HPI) was used as the long‐chain ITF, and oligofructose‐enriched inulin (OEI) was used as the short‐chain ITF. Participants in the intervention groups were provided with 10 g/day HPI (Frutafit® TEX, Sensus, Borchwerf 3, 4704 RG, Roosendaal, Netherlands, DP ≥ 23) (*n* = 25) or 10 g/day OEI (Frutafit® IQ, Sensus, Borchwerf 3, 4704 RG, Roosendaal, Netherlands, DP = 8–13) (*n* = 25). Participants in the placebo group received 10 g/day maltodextrin (Zarfructose Company, Tehran, Iran, Dextrose Equivalent [DE] = 14) (*n* = 25). The intervention duration was 12 weeks. Intervention and placebo materials were provided in similar 10‐g packages in powder, similar in appearance, smell, color, texture, and taste. The dose of 10 g was chosen based on the literature, considering the amounts of ITFs sufficient to promote beneficial changes in the gut microbiota (Wang et al., 2019) but low enough to minimize adverse gastrointestinal side effects. Participants received 45 packages of intervention and placebo materials twice, at baseline and at the follow‐up visit (end of the sixth week). During the first 2 weeks of supplementation, to facilitate gastrointestinal adaptation to the fiber and decrease side effects, participants were instructed to take half of the package (~5 g) in the morning and another half at night. They were instructed to add the powder to food or drinks (water, tea, coffee, or lemon juice) and consume it before or with their meal, and return empty or unused packages to measure compliance. Participants received a weekly phone call to monitor their adherence and ask about possible adverse effects. Participants were also instructed not to make any changes in their usual diet, exercise, or medication during the intervention and to refrain from consuming any types of prebiotics, symbiotics, or probiotic supplements or foods.

### 2.4 Assessment of variables

#### 2.4.1 Dietary intake and physical activity levels

Participants completed a 3‐day food log, including two weekdays and one weekend day, and a physical activity (PA) questionnaire at baseline and the end of the study, with the help of a trained nutritionist, to verify that they maintained their usual diet and physical activity levels during the trial. The recorded food items and drinks were converted to grams/day using standard Iranian household measures (Ghaffarpour et al., 1999). Dietary intakes were then analyzed using the Nutritionist‐4 software (First Databank Inc., San Bruno, CA), which was modified for Iranian foods. The Iranian version of the International Physical Activity Questionnaire (IPAQ) was used to measure and report PA levels as metabolic‐equivalent hours per day (MET/h/day) (Moghaddam et al., 2012).

#### 2.4.2 Anthropometric and blood pressure measurements

Body weight was measured without shoes and with minimal clothing and by a digital Seca scale (Seca 831, Hamburg, Germany), to the nearest 0_·_1 kg. Height was measured in a standing position without shoes by a portable stadiometer (Seca, Hamburg, Germany) to the nearest 0.5 cm. BMI was calculated as body mass divided by height squared (kg/m^2^). A flexible measuring tape was utilized to measure waist circumference (WC) to the precision of 0.1 cm at the midpoint between the ribs and iliac crest. Blood pressure was measured at baseline and at the end of the trial with a conventional mercury sphygmomanometer after participants were seated and rested for 10 min. Blood pressure was measured twice with at least a 30‐s interval between measures, and the average of the two measurements was used for the analyses.

### 2.5 Assessment of PCOS clinical features

Each participant's hirsutism score and menstrual cycle characteristics were assessed at baseline and at the end of the trial by a trained researcher (R.Z). Menstrual cycle characteristics, including menstrual cycle length and abnormalities of the menstrual cycle, were assessed using an interview and a checklist designed for this study. Women with menses every 21–34 days were considered to have regular menstruation; otherwise, irregular menstruation was recorded (Klein et al., 2019). Secondary amenorrhea was characterized by the cessation of previously regular menses for 3 months or previously irregular menses for 6 months (Klein et al., 2019). Oligomenorrhea was characterized as a menstrual cycle length greater than 35 days, or four to nine menstrual cycles in a year (Azziz, 2018). For evaluating hirsutism, the modified Ferriman‐Gallwey (mFG) scoring system was used, and 9 body areas, including upper abdomen, lower abdomen, thighs, back, arm, upper lip, chin, chest, and buttocks, were examined for hair and scored from 0 (no visible terminal hair) to 4 (terminal hair growth with a male pattern) (Hatch et al., 1981). The total scores range from 0 to 36.

### 2.6 Main outcomes

Markers of IR [serum fasting insulin level, Homeostatic Model Assessment (HOMA‐IR), and Quantitative Insulin‐Sensitivity Check Index (QUICKI)], hormonal status [total testosterone, sex hormone‐binding globulin (SHBG), and Free Androgen Index (FAI)], as well as clinical variables [hirsutism and menstrual cycle status] were considered primary outcomes. Other variables, such as serum lipid levels, fasting plasma glucose (FPG), and anthropometric indices, were considered secondary outcomes.

### 2.7 Randomization and blinding

The participants were randomly allocated using a stratified permuted block randomization design (with block size 3) to one of the 3 treatment groups (A, B, or C) by a random number generator (https://www.sealedenvelope.com/simple‐randomiser/v1/lists) to create a blocked randomization list, in which A was the HPI group, B was the OEI group, and D was the placebo group. Participants were stratified based on age (18–30 years and 30–40 years) and groups (HPI, OEI, and placebo). A trained person who was not involved in the selection and randomization of the participants prepared, coded, and sealed the opaque packages. Participants, laboratory staff, and investigators were blinded to the treatment allocation until the main analyses were completed.

### 2.8 Biochemical assessment

Fasting blood samples (10 mL) were collected at baseline and the end of the trial by a trained phlebotomist and assessed at one of the reference laboratories in Isfahan. Samples were centrifuged for 10 min at 2500 rpm (Beckman Avanti J‐25; Beckman 174 Coulter, Brea, CA, USA) at room temperature. FPG and serum lipid concentrations were analyzed on the day of sampling, and the remaining serum was stored at −80°C until the assays were complete. FPG, serum triglycerides, total cholesterol, and LDL‐ and HDL‐cholesterol concentrations were measured using the enzymatic colorimetric assay (Pars Azmun, Tehran, Iran). All inter‐ and intra‐assay coefficients of variability (CVs) for FPG and blood lipid measurements were less than 5%. Serum fasting insulin levels were assessed using an ELISA kit (Monobind, California, USA) with intra‐ and inter‐assay CVs of 4.8%. HOMA‐IR, as a measure of IR, was calculated using the following formula: HOMA‐IR = (fasting insulin [mIU/L] × fasting blood glucose [mg/dL])/405 (or (fasting insulin [mIU/L] × fasting blood glucose [mmol/L])/22 × 5) (Albareda et al., 2000). For assessing the degree of insulin sensitivity, the QUICKI was determined using the following formula: 1/(log fasting glucose + log fasting insulin) (Katz et al., 2000). Serum total testosterone and SHBG levels were measured using a validated commercial kit (DiaMetra, Milano, Italy), with inter‐ and intra‐assay CVs of less than 7%. The free androgen index (FAI) was calculated as the percentage of total testosterone to SHBG.

### 2.9 Sample size

The sample size was calculated using the formula for a randomized controlled trial with a three‐arm parallel design. Considering the effect size reported in similar interventions (Dehghan et al., 2013; Ziaei et al., 2022), a standard effect size of 0.9 was estimated for the main outcomes to detect significant differences between groups. Considering *g* = 3, *α* = 0.05, power = 80%, and the standard effect size ≥0.9, a total of 75 subjects was required.

n1=1+g−1z1−α2+z1−β2∆2+z1−α22g−121+g−1=1+3−17.8490.92+1.9623−121+3−1≅25

$$n=n_{1}+\left(g-1\right)n_{2}=25+\left(2*25\right)=75$$

### 2.10 Statistical methods

Statistical analyses were performed with SPSS software (IBM SPSS Statistics for Windows version 22.0; IBM Corp.). Data were evaluated and managed for the presence of missing data and violations of normality. Q–Q plots, skewness statistics, and the Shapiro–Wilk test were used for assessing the normal distribution of variables. Data were reported as mean ± standard deviations (SD) for continuous variables and frequency with percentages for categorical variables. Appropriate transformation approaches were applied for variables with an abnormal distribution. One‐way ANOVA and Chi square analyses were used to compare the baseline characteristics of study participants between groups. An analysis of covariance (ANCOVA) with the adjustment of baseline values and potential covariates, including weight change and use of insulin sensitizers, was used to compare continuous variables post‐intervention. Bonferroni post‐hoc analysis was used for two‐by‐two comparisons of the groups, where appropriate. Paired *t‐*tests or the McNemar test (for categorical data) were used to compare outcome variables pre‐ and post‐intervention. To estimate the effect of the intervention on binary variables, a logistic regression analysis adjusted for confounding variables was performed. For intent‐to‐treat analysis (ITT), missing values were imputed using the regression‐based multiple imputation procedure. A *p*‐value <.05 was considered statistically significant.

## 3 RESULTS

**FIGURE 1:**
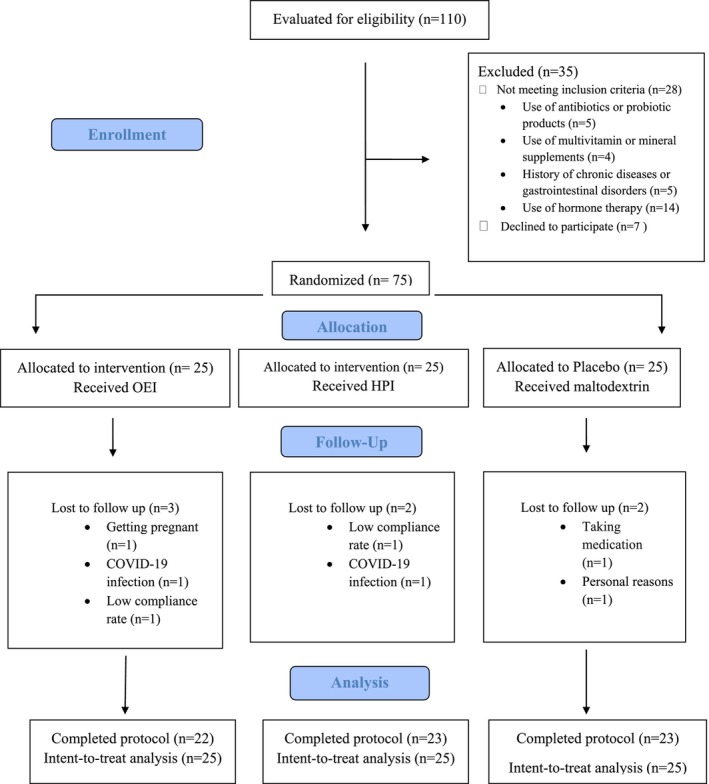
Flow diagram of study recruitment.

A total of 110 women with PCOS were screened, of whom 75 met the inclusion criteria and consented to participate. Seven patients were lost to follow‐up, including three in the HPI group (due to getting pregnant, *n* = 1; low compliance rate, *n* = 1; and a COVID‐19 infection, *n* = 1), two in the OEI group (a COVID‐19 infection, *n* = 1 and low compliance rate, *n* = 1), and two in the placebo group (personal reasons, *n* = 1 and taking medication, *n* = 1) (Figure 1). Sixty‐eight women completed the 12‐week intervention; however, all 75 participants were included in the ITT analyses.

Mild to moderate gastrointestinal issues were reported during the first 2 weeks of intervention in three participants from the HPI group (12%) and one in the OEI group (4%), including mild bloating (*n* = 2), abdominal cramps (*n* = 1), and nausea (*n* = 1). However, these complications were transient and improved in the following weeks.

Baseline characteristics of participants, including mean age, height, body weight, BMI, physical activity level, blood pressure, marital status, history of infertility, menstrual cycle characteristics, and use of insulin sensitizers, were similar between the HPI, OEI, and placebo groups (Table 1). No significant differences were observed between the three groups in baseline nutritional intakes (mean energy, macronutrients, dietary fat, total dietary fiber, and cholesterol intakes). The nutritional intakes of the women were not different before and after treatment between HPI, OEI, and placebo groups. Details of the dietary intakes of study participants were presented in the previous published work from the same research project (Ziaei et al., 2022).

**TABLE 1:** General characteristics of study participants.

	Groups			*p* value
	HP inulin (*n* = 25)	OE inulin (*n* = 25)	Placebo (*n* = 25)	
Age (years)	29.4 ± 5.5	28.9 ± 5.1	28.8 ± 5	.89^a^
Height (cm)	162 ± 5.9	162 ± 5.4	162 ± 4.4	.99^a^
Weight at study baseline (kg)	76.49 ± 12.67	74.51 ± 12.92	75.66 ± 12.81	.795^a^
BMI at study baseline (kg/m^2^)	28.77 ± 3.6	27.95 ± 3.63	28.54 ± 4.25	.69^a^
SBP	109.13 ± 12.3	109.16 ± 12.89	106.2 ± 12.53	.72^a^
DBP	74.34 ± 7.27	71.25 ± 8.37	73.33 ± 12.39	.87^a^
MET‐h/day at study baseline	24.2 ± 18.5	26.1 ± 17.9	22.08 ± 10.1	.66^a^
MET‐h/day at end‐of‐trial	23.8 ± 17.03	25.9 ± 16.8	21.8 ± 10.3	.64^a^
MET‐h/day change	−1.5 ± 5.1	0.62 ± 2.9	−0.33 ± 2.6	.13^a^
Marital status				
Single	3 (12)	5 (20)	6 (24)	.76^b^
Married	21 (84)	18 (72)	17 (68)	
Divorced	1 (4)	2 (8)	2 (8)	
Occupation				
Housewife	14 (56)	12 (48)	18 (72)	.22^b^
Employed	10 (40)	11 (44)	4 (16)	
Student	1 (4)	2 (8)	3 (12)	
Education level				
Less than a high school diploma	0	0	2 (8)	.34^b^
High school diploma	22 (88)	23 (92)	20 (80)	
University education	3 (12)	2 (8)	3 (12)	
History of infertility				
Yes	13 (52)	8 (32)	13 (52)	.26^b^
No	12 (48)	17 (68)	12 (48)	
Diagnosis period (years)	6.9 ± 5.6	5.1 ± 5.3	6.2 ± 5.2	.5^a^
Menstruation				
Regular	10 (40)	7 (28)	12 (48)	.34^b^
Irregular	15 (60)	18 (72)	13 (52)	.34^b^
Oligomenorrhoea	9 (36)	9 (36)	8 (32)	.94^b^
Amenorrhea	5 (20)	8 (32)	3 (12)	.22^b^
Insulin sensitizers				
Yes	16 (64)	9 (36)	10 (40)	.1^b^
No	9 (36)	16 (64)	15 (60)	

*Note*: Data are mean ± SD or number (percentage).

Abbreviations: BMI, body mass index; MET, metabolic equivalent of task.

^a^ Obtained from One‐Way ANOVA.

^b^ Obtained from Chi‐squared test.

### 3.1 Main outcome measures

After 12 weeks of supplementation, WC decreased significantly in both HPI and OEI groups (mean difference [MD]: −3.47 and −2.12 for the HPI and OEI groups, respectively, *p* < .001), but not in the placebo group. Body weight (MD in the HPI group: −2.86 vs. MD in the placebo group: −0.43; *p* < .001) and BMI (MD in the HPI group: −1.05 vs. MD in the placebo group: −0.15; *p* < .001) decreased in the HPI and OEI groups, but the changes were only significant in the HPI group when compared with the placebo group. A post‐hoc test using the Bonferroni correction determined a significant difference between HPI and OEI with regard to body weight and BMI (MD: −1.73; *p* = .001 and MD: −0.61; *p* = .003 for body weight and BMI, respectively) (Table 2).

**TABLE 2:** Anthropometric parameters, hirsutism score, metabolic and endocrine profiles of the placebo, high‐performance inulin, and oligofructose‐enriched inulin groups at baseline and after the 12‐week intervention.

Variable	HPI		OEI		Placebo		*p* ^b^	*p* ^c^	Post‐hoc group^d^
	Mean ± SD	MD	Mean ± SD	MD	Mean ± SD	MD			
Weight (kg)									
Before	76.49 ± 12.67	−2.86 ± 2.41	74.51 ± 12.92	−1.13 ± 1.1	75.66 ± 12.81	−0.43 ± 1.14	<.001	<.001	A/B A/C
After	73.63 ± 11.52		73.38 ± 12.76		75.22 ± 12.93				
*p* ^a^	<.001		<.001		.07				
BMI (kg/m^2^)									
Before	28.77 ± 3.6	−1.05 ± 0.81	27.95 ± 3.63	−0.42 ± 0.43	28.54 ± 4.25	−0.15 ± 0.43	<.001	<.001	A/B A/C
After	27.71 ± 3.44		27.53 ± 3.58		28.38 ± 4.32				
*p* ^a^	<.001		<.001		.07				
WC (cm)									
Before	94.78 ± 9.54	−3.47 ± 1.92	91.7 ± 9.36	−2.12 ± 1.87	91.39 ± 8.15	−0.56 ± 1.87	<.001	<.001	A/C B/C A/B
After	91.3 ± 9.15		89.58 ± 8.86		91.6 ± 7.9				
*p* ^a^	<.001		<.001		.16				
FPG (mg/dL)									
Before	90.82 ± 12.71	−2.69 ± 6.59	90.12 ± 13.47	−2.66 ± 7.21	89.54 ± 11.68	−0.79 ± 4.89	.46	.53	‐
After	88.13 ± 9.23		87.45 ± 8.97		88.75 ± 12.57				
*p* ^a^	.06		.08		.43				
FI (micro IU/mL)									
Before	14.44 ± 6.49	−3.23 ± 3.98	13.03 ± 5.84	−1.83 ± 4.08	13.27 ± 5	−0.25 ± 3.22	.01	.01	A/C
After	11.21 ± 5.15		11.19 ± 3.92		13.01 ± 4.51				
*p* ^a^	.001		.03		.69				
HOMA‐IR									
Before	3.36 ± 2.03	−0.87 ± 1.06	2.92 ± 1.48	−0.49 ± 1.04	2.95 ± 1.21	−0.12 ± 0.71	.01	.01	A/C
After	2.49 ± 1.38		2.42 ± 0.9		2.83 ± 0.97				
*p* ^a^	.001		.02		.41				
QUICKI									
Before	0.32 ± 0.02	0.01 ± 0.01	0.33 ± 0.02	0.006 ± 0.01	0.33 ± 0.022	0.00006 ± 0.01	.008	.01	A/C
After	0.34 ± 0.02		0.33 ± 0.01		0.33 ± 0.017				
*p* ^a^	<.001		.02		.98				
Triglycerides (mg/dL)									
Before	165.26 ± 106.75	−37.04 ± 42.7	141.41 ± 52.67	−28.04 ± 27.1	156.41 ± 22.03	−4.66 ± 4.81	<.001	<.001	A/C B/C
After	128.21 ± 78.04		113.37 ± 42.11		151.75 ± 21.38				
*p* ^a^	.001		<.001		.34				
Total cholesterol (mg/dL)									
Before	181.39 ± 48.53	−9.65 ± 24.6	171.41 ± 43.15	−5.7 ± 7.6	158.75 ± 32.07	0.79 ± 8.4	.37	.49	‐
After	171.73 ± 49.4		165.7 ± 35.91		159.54 ± 30.7				
*p* ^a^	.07		.12		.64				
LDL cholesterol (mg/dL)									
Before	99.86 ± 40.8	−5.3 ± 22.2	97.45 ± 32.8	−6.58 ± 9.5	84.08 ± 26.5	−0.04 ± 8.34	.81	.95	‐
After	94.56 ± 30.3		90.87 ± 31.07		84.04 ± 22.3				
*p* ^a^	.26		0.11		.98				
HDL cholesterol (mg/dL)									
Before	48.04 ± 11.6	1.26 ± 8.88	50.45 ± 14.5	2 ± 9.33	54.12 ± 12.7	−1.7 ± 5.93	.42	.35	‐
After	49.3 ± 10.8		52.45 ± 15.6		52.41 ± 11.9				
*p* ^a^	.5		.3		.17				
LDL‐C/HDL‐C ratio									
Before	2.27 ± 1.23	−0.23 ± 0.51	2 ± 0.65	−0.08 ± 0.52	1.68 ± 0.84	0.08 ± 0.3	.31	.34	‐
After	2.04 ± 1		1.91 ± 0.63		1.77 ± 0.8				
*p* ^a^	.04		.43		.16				
Total Testosterone (ng/mL)									
Before	1.07 ± 0.5	−0.27 ± 0.23	1.09 ± 0.35	−0.06 ± 0.11	1.06 ± 0.42	0.04 ± 0.12	<.001	<.001	A/B A/C B/C
After	0.8 ± 0.4		1.02 ± 0.32		1.1 ± 0.37				
*p* ^a^	<.001		.01		.11				
SHBG (nmol/L)									
Before	34.43 ± 18.19	9.73 ± 6.96	34.95 ± 18.1	9.29 ± 6.42	38.37 ± 16.38	−1.7 ± 6.72	<.001	<.001	A/C B/C
After	44.17 ± 15.1		44.25 ± 17.5		36.66 ± 14.59				
*p* ^a^	<.001		<.001		.22				
FAI									
Before	4.23 ± 3.29	−2.11 ± 2.45	4.07 ± 2.67	−1.34 ± 1.41	3.45 ± 2.47	0.12 ± 0.89	<.001	<.001	A/C B/C
After	2.11 ± 1.34		2.72 ± 1.55		3.58 ± 2.29				
*p* ^a^	<.001		.001		.49				
mF‐G scores									
Before	14.09 ± 8.84	−2.52 ± 1.95	13.58 ± 9.11	−1.37 ± 1.83	12.58 ± 8.18	−0.5 ± 0.42	.001	.001	A/C A/B
After	11.57 ± 7.78		12.21 ± 8		12.08 ± 7.54				
*p* ^a^	<.001		.02		.25				
Menstrual cycle length									
Before	41.7 ± 21.6	−7.33 ± 14.5	40.81 ± 19.19	−6.12 ± 14.7	37.05 ± 13.46	0.9 ± 10.4	.07	.07	‐
After	34.38 ± 9.86		34.68 ± 9.19		37.95 ± 12.68				
*p* ^a^	.04		.11		.7				

*Note*: Conversion factors to the International System of Units (SI): FPG (mg/dL to mmol/L) = 0/05551; FI (microIU/mL to pmol/L) = 7/175; TG (mg/dL to mmol/L) = 0/011; TC (mg/dL to mmol/L) = 0/0258; LDL‐C (mg/dL to mmol/L) = 0/0258; HDL‐C (mg/dL to mmol/L) = 0/0258; Total Testosterone (ng/mL to nmol/L) = 3/467.

Abbreviations: BMI, body mass index; FAI, free androgen index; FI, fasting insulin; FPG, fasting plasma glucose; SHBG, sex hormone‐binding globulin; WC, waist circumference.

^a^ *p*‐Value was reported based on paired sample *t*‐test.

^b^ *p*‐Value was reported based on ANCOVA (adjusted baseline value).

^c^ *p*‐Value was reported based on ANCOVA (adjusted baseline value, use of insulin sensitizers, and baseline BMI). *p* < .05 = statistically significant.

^d^ Based on post‐hoc test using the Bonferroni correction. A represents HPI group, B represents OEI group, and C represents placebo group.

At the end of the trial, the serum fasting insulin level (MD in HPI group: −3.23 vs. MD in placebo group: −0.25 μIU/mL [conversion factor to SI units (pmol/L): 7/175]; *p* = .02) and HOMA‐IR (MD in HPI group: −0.87 vs. MD in placebo group: −0.12; *p* = .01) decreased in the HPI and OEI groups, but the changes were only significant for HPI compared to placebo. Also, QUICKI increased significantly by the end of the intervention in the HPI group when compared with the placebo group (MD in the HPI group: 0.01 vs. MD in the placebo group: 0.00006; *p* = .01). The FPG level was not found to be different between or within the three groups (Table 2).

Total testosterone levels (MD in the HPI group: −0.27 vs. MD in the OEI group: −0.06 vs. MD in the placebo group: 0.04 ng/mL [conversion factor to SI units (nmol/L): 3/467]; *p* < .001) and FAI (MD in the HPI group: −2.11 vs. MD in the OEI group: −1.34 vs. MD in the placebo group: 0.12; *p* < .001) decreased in the HPI and OEI groups over the 12‐week intervention, and the changes were significant in both groups compared to the placebo. Also, a significant increase in serum SHBG concentrations was observed in both the HPI and OEI groups compared to the placebo (MD in the HPI group: 9.73 vs. MD in the OEI group: 9.29 vs. MD in the placebo group: −1.7 nmol/L; *p* < .001) (Table 2).

Regarding clinical manifestations of PCOS, the hirsutism score decreased in the HPI and OEI groups, and the changes were significant in the HPI group compared to the placebo (MD in the HPI group: −2.52 vs. MD in the placebo group: −0.5; *p* < .001) at the end of the trial. Based on post‐hoc analyses, changes in total testosterone levels and hirsutism scores were higher among the HPI group than the OEI group (MD: −0.21; *p* < 0.001 and MD: −1.08; *p* = .04, respectively) (Table 2).

As shown in Table 2, there was not a significant difference between the three groups in terms of menstrual cycle length.

Data regarding menstrual abnormalities is shown in Table 3. The number of women with irregular menstrual cycles (*p* = .004) and oligomenorrhoea (*p* = 0.005) decreased, and the number of women with regular menstruation (*p* = 0.004) increased significantly in the HPI and OEI groups compared with the placebo. Amenorrhea was not found to be different between or within the three groups.

**TABLE 3:** A comparison of menstrual cycle status between the placebo, high‐performance inulin, and oligofructose‐enriched inulin groups at baseline and after the 12‐week intervention.

Menstrual cycle status	HPI	OEI	Placebo	*p* ^b^	*p* ^c^
	*N* (%)	*N* (%)	*N* (%)		
Regular					
Before	10 (40)	7 (28)	12 (48)	.005	.004
After	14 (56)	13 (52)	7 (28)		
*p* ^a^	0.031	0.031	0.219		
Irregular					
Before	15 (60)	18 (72)	13 (52)	.005	.004
After	9 (36)	11 (44)	17 (68)		
*p* ^a^	.031	.031	.219		
Oligomenorrhea					
Before	9 (36)	9 (36)	8 (32)	.004	.005
After	4 (16)	4 (16)	12(48)		
*p* ^a^	.063	.125	.219		
Amenorrhea					
Before	5 (20)	8 (32)	3 (12)	.853	.824
After	5 (20)	7 (28)	3 (12)		
*p* ^a^	1	1	1		

^a^ *p*‐Value was reported based on McNemar Test.

^b^ *p*‐Value was reported based on Logistic regression (adjusted baseline value).

^c^ *p*‐Value was reported based on Logistic regression (adjusted baseline value, use of insulin sensitizers, baseline BMI). *p* < .05 = statistically significant.

Twelve weeks of supplementation with HPI and OEI induced a significant decrease in mean serum levels of triglycerides compared to the placebo (MD in the HPI group: −37.04 vs. MD in the OEI group: −28.04 vs. MD in the placebo group: −4.66 mg/dL [conversion factor to SI units (mmol/L): 0/011]; *p* < .001). However, the three groups showed no difference in mean serum levels of total cholesterol, LDL‐C, HDL‐C, and the LDL‐C/HDL‐C ratio. Post‐intervention levels of serum triglyceride were not significantly different between the HPI and OEI groups (MD: −4.88; *p* > .05) (Table 2).

## 4 DISCUSSION

The aim of this study was to investigate and compare the effects of inulin supplementation with different DP on anthropometric and metabolic parameters, IR, androgen status, and clinical manifestations in women with PCOS. We found that both HPI and OEI supplementations for 12 weeks had beneficial effects on weight loss, serum total testosterone, FAI, serum SHBG, serum triglycerides, and clinical manifestations of PCOS, including menstrual abnormalities, but they did not significantly affect FPG and other serum lipid parameters. Also, it was concluded that the beneficial effects of HPI on body weight, BMI, fasting insulin, IR, and the hirsutism score are more remarkable than OEI.

Although obesity is not the leading cause of PCOS, it exacerbates IR as well as metabolic and reproductive disorders associated with the disease (Cena et al., 2020). We found that supplementation with ITFs for 12 weeks, especially those with higher DP, had favorable effects on body weight, BMI, and WC in women with PCOS. To our knowledge, this study is the only trial assessing the effects of ITFs on metabolic and hormonal measures in PCOS patients; however, inulin supplementation was associated with beneficial anti‐obesity effects in other target populations (Visuthranukul et al., 2022). In a study by Guess et al. (2015), inulin supplementation for 18 weeks promoted weight loss and reduced hepatic and soleus muscle fat content, independent of weight loss, in prediabetic subjects. Interest regarding the potential role of gut dysbiosis in the homeostasis of energy metabolism and obesity has grown dramatically in recent years. The anti‐obesogenic impact of ITFs may be partly attributed to SCFAs, which promote energy expenditure and fatty acid oxidation in the mitochondria of the liver and muscles via the activation of 5′‐AMP‐activated protein kinase (Amabebe et al., 2020). Also, SCFAs exhibit anti‐inflammatory properties by promoting gut barrier function, thus favorably affecting obesity (Martin‐Gallausiaux et al., 2021). Moreover, ITFs induce satiety and reduce food intake through increased secretion of anoretic hormones such as glucagon‐like peptide‐1 (GLP‐1) in animal and human studies (Alptekin et al., 2022; Hiel et al., 2019).

Insulin resistance may be involved in the three classical features and metabolic defects associated with PCOS (He & Li, 2020; Zeng et al., 2020). So, targeting IR may be an effective approach to the management of PCOS. We found a significant improvement in insulin sensitivity, following supplementation with HPI for 12 weeks. The beneficial effect of inulin on IR was irrespective of obesity, as findings remained significant after further adjustments for body weight. Our findings are in agreement with several previous studies that have been conducted in the field; however, these studies were conducted among different target populations. A meta‐analysis by Rao et al. (2019) reported a significant reduction in IR in patients with type‐2 diabetes melitus (T2DM) following supplementation with ITFs. Insulin resistance is interrelated with the intestinal microbiota, and gut dysbiosis is more notable in insulin‐resistant PCOS patients (He & Li, 2020). Increased intestinal permeability following an imbalance of gut microbiota causes low‐grade inflammation, which interferes with the function of insulin receptors (Dong et al., 2023; Zeng et al., 2019). Several mechanisms were proposed to explain the beneficial effects of ITFs on IR and glycemic control, such as improving gut integrity due to SCFA production (Birkeland et al., 2020; Li et al., 2019), enhancing the production of peptide YY and GLP‐1, and promoting weight loss (Catry et al., 2018; Morrison & Preston, 2016).

The FPG level was not found to be different between or within the three groups in the present study. The findings regarding the blood glucose‐lowering impact of ITFs are contradictory. Several studies, mainly conducted in diabetic or prediabetic populations, have reported a significant reduction in blood glucose concentrations following inulin supplementation (Li et al., 2021; Zhang et al., 2020). However, Liu et al. (2017), in a meta‐analysis of randomized controlled trials on the effect of ITFs on glycemic control, observed a reduced FPG level among the T2DM subgroup only. We did not include PCOS women who were diabetic or prediabetic in the present study. Patients with diabetes might be more responsive to the intervention due to their different intestinal bacterial composition and elevated insulin and glucose levels.

Hyperandrogenemia aggravates IR through increased formation of free fatty acids (FFAs) and decomposition of visceral adipose tissue (He & Li, 2020; Pateguana & Janes, 2019). Since hyperinsulinemia promotes androgen production, a vicious cycle promoting hyperandrogenism and IR is formed in PCOS. Inulin supplementation induced a significant reduction in serum total testosterone concentrations and the hirsutism score as the main clinical symptoms of hyperandrogenism. We considered FAI as a valid estimate of true androgen status (Blight et al., 1989), and FAI decreased significantly in both HPI and OEI groups. Due to the lack of similar studies in this field, we compared our findings with those evaluating the effect of other prebiotics, synbiotics, or probiotics on hormonal status in PCOS, and we found our findings to be consistent with these (Cozzolino et al., 2020; Hadi et al., 2020). Shamasbi et al. (2020), in a meta‐analysis on the effects of probiotics, prebiotics, and synbiotics on hormonal status in PCOS, showed a significant increase in SHBG and a significant decrease in FAI following treatment.

The number of patients experiencing irregular menses, including both polymenorrhea and oligomenorrhea, was reduced in HPI and OEI groups. Inulin supplementation did not affect the number of patients with amenorrhea, which might be due to the short duration of our study. Menstrual cycle length was not found to be different between the three groups. Prebiotics such as ITFs inhibit inflammation and attenuate hyperinsulinemia (Yurtdaş & Akdevelioğlu, 2020). Hyperinsulinemia leads to hyperandrogenism and disrupts normal follicular growth, as the severity of hirsutism and menstrual abnormalities is positively correlated with the degree of hyperinsulinemia (Ezeh et al., 2022).

Our results regarding the triglyceride‐lowering effect of ITFs are in line with several animal and human studies (Hiel et al., 2018; Li et al., 2021). Despite the nonsignificant effects of inulin supplementation on other lipid endpoints, we observed a minimally significant improvement in the LDL‐C/HDL‐C ratio following HPI supplementation based on within‐group analyses. Guo et al. (2012) conducted a meta‐analysis to evaluate and compare the effects of ITFs on the blood lipids of normolipidemic and hyperlipidemic subjects. They found inulin to beneficially affect serum triglyceride, TC, and LDL‐C levels in hyperlipidemic participants but not in normolipidemic participants. Our study participants had relatively normal cholesterol values at baseline, which might partly explain our nonsignificant findings in this regard. The lipid‐lowering mechanisms of ITFs have not been fully elucidated. Increased fecal bile acid excretion as well as the formation of SCFAs, which inhibit cholesterol synthesis and promote weight loss, are among the proposed mechanisms in this field (Dong et al., 2022; Hughes et al., 2022; Qin et al., 2023).

ITFs with different DPs induce different effects on the gut microbiome due to their different fermentation patterns (Astó et al., 2019). Based on several animal and in vitro studies, the modulating effects of long‐chain ITFs on gut microbiota, formation of SCFAs, metabolic endotoxemia and inflammation, and glucose and lipid homeostasis were more pronounced than those of short‐chain ITFs (Astó et al., 2019; Li et al., 2020). However, a few studies reported contradictory results (Ruan et al., 2019). Little information exists on the impact of fructans with different DPs on gut dysbiosis and metabolic defects in humans. Liu et al. (2017), in a meta‐analysis on the effects of ITFs on blood lipids and glucose levels, conducted a subgroup analysis of the inulin (DP ≥ 10) and FOS (DP < 10) intervention trials. They found inulin, but not FOS, beneficially affects blood lipid concentrations. ITFs with higher DP induce higher alpha‐diversity as well as SCFA production and acidification activity, compared with those with lower DP (Astó et al., 2019). Also, their fermentation takes a longer time, making their prebiotic activity in the distal part of the colon more prominent than short‐chain inulin.

The present study is the first to investigate the effects of ITFs on the metabolic and hormonal parameters of women with PCOS in a randomized, controlled trial with a relatively long duration. Also, in this study, we differentiate and compare the effects of fructans with different DP on various parameters in PCOS patients for the first time. However, this study had some limitations. First, gut bacterial changes, SCFA production, and gut epithelial permeability were not assessed before and after the intervention to confirm the modulating impact of ITFs on gut dysbiosis. Additionally, patient compliance is not fully known. Analyzing intestinal bacterial changes should be a point of focus in future studies. Furthermore, for some variables, including the LDL‐C/HDL‐C ratio and menstrual cycle length, a larger sample size might be required to obtain statistical significance. Moreover, since we only recruited overweight and obese women with PCOS, our findings cannot be generalized to all PCOS patients. Other limitations include not measuring body composition changes and the fact that self‐reported evaluations of dietary intakes were used.

## 5 CONCLUSION

A 12‐week supplementation with either HPI or OEI may improve visceral obesity, hormonal status, serum triglyceride level, and menstrual cycle irregularities in overweight and obese women with PCOS. Also, ITFs with higher DP appear to be more powerful at improving obesity measures, IR, and the hirsutism score. Overall, ITFs might be considered a novel therapeutic approach for patients with PCOS due to their modifying effects on IR and hyperandrogenemia. Further studies with larger sample sizes and fewer limitations are needed to confirm our findings. Gut bacterial changes, SCFA production, and gut epithelial permeability should be assessed in future studies to confirm the relationship between ITF supplementation and PCOS.

## AUTHOR CONTRIBUTION

**Rahele Ziaei:** Conceptualization (equal); data curation (equal); formal analysis (equal); methodology (lead); resources (equal); software (equal); supervision (equal); writing – original draft (lead); writing – review and editing (equal). **Zahra Shahshahan:** Investigation (equal). **Hatav Ghasemi‐Tehrani:** Investigation (equal); methodology (equal). **Zahra Heidari:** Data curation (equal); formal analysis (equal); software (equal). **Marilyn S. Nehls:** Writing – review and editing (equal). **Reza Ghiasvand:** Conceptualization (equal); funding acquisition (equal); writing – review and editing (equal).

## FUNDING INFORMATION

The present study was supported by a grant from the Vice‐Chancellor for Research of the Isfahan University of Medical Sciences (Grant no: 399461).

## CONFLICT OF INTEREST STATEMENT

The authors declare no conflicts of interest.

## TRANSPARENCY DECLARATION

The corresponding author (R.GH) affirms that this manuscript is an honest, accurate, and transparent account of the study being reported. The reporting of this work is compliant with CONSORT guidelines. No important aspects of the study have been omitted and any discrepancies from the study as planned have been disclosed.

## Data Availability

Data available upon request.
